# PIM2 induced MMP-9 expression in macrophages requires PI3K and Notch1 signaling

**DOI:** 10.1186/1479-5876-10-S3-P23

**Published:** 2012-11-28

**Authors:** Nisha Kapoor, Kushagra Bansal, Germain Puzo, Martine Gilleron, KN Balaji

**Affiliations:** 1 Dept. of Microbiology and Cell Biology, Indian Institute of Science, Bangalore, India; 2 Dept. of Mechanisms of Mycobacterial Infections, Université Paul Sabatier, Toulouse, France

## Introduction

Granuloma formation during *Mycobacterium tuberculosis* infection represents pathological attributes of the host immunity to infection and is required for the containment of infection. Granuloma formation is a complex process involving initiation and development of organized multicellular structures comprised of components of extracellular matrix [1]. Activation of inflammatory immune responses during granuloma formation upon infection with mycobacteria is often associated with tissue remodeling and breakdown of the extracellular matrix. In these complex processes, Cyclooxygenase-2 plays a major role in chronic inflammation and regulates matrix metalloproteinase-9 expression significantly in tissue remodeling but the molecular mechanisms involved remain elusive.

## Aim

To investigate the molecular mechanisms underlying Phosphatidyl-myo-inositol dimannosides triggered MMP-9 expression in macrophages.

## Methods

We show possible implications of Notch signaling on immunological parameters associated with interaction of macrophages with novel cell wall antigen of Mycobacteria. We present evidences that PIM2 triggered expression of MMP-9 involves the activation of PI3K and Notch1 signaling in TLR2- MyD88 dependent manner.

## Results

PIM2 triggers the activation of PI3K and Notch1 signaling leading to MMP-9 expression. Notch1 signaling perturbations demonstrate the involvement of a cross-talk with members of PI3K and MAPK pathway. PIM2 triggered significant p65 NF-κB nuclear translocation that was dependent on activation of PI3K or Notch1 signaling. MMP-9 expression requires Notch1 mediated recruitment of Suppressor of Hairless (CSL) and NF-κB to respective promoters.

## Conclusions

PI3K and Notch1 signaling are obligatory early proximal signaling events during PIM2 induced MMP-9 expression in macrophages.

**Figure 1:**
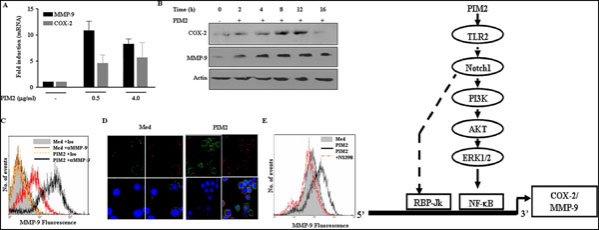
PIM2 induced COX-2 and MMP-9 expression and a model depicting signaling cascades regulating their expression: PIM2 treated macrophages shows upregulated COX-2 and MMP-9 expression both at mRNA and protein levels as analyzed by qRT-PCR, immunoblotting, FACS and confocal microscopy. Immunoflourescent staining of NS-398 treated macrophages shows decreased MMP-9 expression.
